# Omental patch to control hepatic exsanguination

**Published:** 2012-08-10

**Authors:** Stephen Apanga, Kenneth V Iserson, Damien Punguyire

**Affiliations:** 1 Kintampo Health Research Centre and Kintampo Municipal (District) Hospital, Kintampo, Ghana; 2 The University of Arizona, Tucson, AZ, USA; 3 Kintampo Municipal Hospital, Kintampo, Ghana

**Keywords:** Omental patch, Intraperitoneal hemorrhage, Hepatic cancer

## Abstract

Acute spontaneous intra-abdominal hemorrhage can be life-threatening and is particularly challenging in resource-poor settings. A 35-year-old woman presented in acutely hypotensive with a distended, rigid abdomen. A paracentesis produced >10 mL of non-clotting blood and she was taken to the operating room where significant bleeding from a liver cancer nodule was identified. With no other option, the generalists doing the surgery used a novel technique - the omental patch - to stem the hemorrhage. The patient recovered from the surgery. The urgency of performing surgery for spontaneous intraperitoneal hemorrhage increases with the scarcity of transfusable blood and general medical officers’ lack surgical experience. In this case, they rapidly improvised, innovatively adapting the omental patch procedure, normally used to close duodenal ulcers, to save a life. They had neither performed nor seen this procedure previously. An omental patch to stop massive localized intra-abdominal hemorrhage may be an important tool for all surgeons.

## Introduction

Acute spontaneous intra-abdominal hemorrhage can be life-threatening under any circumstances. In resource-poor settings, a lack of diagnostic modalities, trained personnel, and therapeutic options challenge any physician's skills. Innovative thinking and rapid action may be the key to saving such patients’ lives.

## Patient and observation

A 35-year-old woman presented at night to the Kintampo Municipal (District) Hospital in acute distress, hypotensive and with a distended abdomen. About 5 hours earlier she had experienced a sudden, severe onset of abdominal pain and distention. The patient denied a history of weight loss, trauma, HIV, ethanol abuse, prior surgery, current medications, menstrual irregularities, and other relevant medical conditions.

On physical exam, she appeared generally healthy with a distended, tender and rigid abdomen without evidence of a mass or ecchymosis. Intravenous crystalloid and then blood was infused. Her hemoglobin was only 7.8 g/dl. Because ultrasound was not immediately available, a paracentesis was done, producing >10 mL of non-clotting blood. Although a stat urine pregnancy test was negative, the most likely preoperative diagnosis was still thought to be a ruptured ectopic pregnancy. With evidence of a massive hemoperitoneum, she was quickly taken to the operating room. She arrived in the operating room hemodynamically stable and with a normal pulse oximetry reading.

### Surgical Procedure

**Figure 1:**
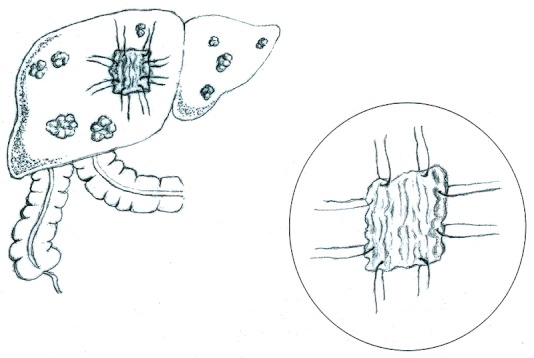
Omental patch on liver lesion

Since an ectopic pregnancy was suspected, a Pfannenstiel incision was made. Finding no evidence of an ectopic pregnancy or other gynecological bleeding, a midline incision was made for better intra-abdominal exposure. A large hemoperitoneum was drained. The entire liver was found to be nodular—apparently from cancer; one of the nodules had ruptured and was identified as the source of the continuing hemorrhage. Another unit of blood (all that was available) was transfused during surgery. One of the two options available (in a hospital with limited resources and operating physicians who were not trained surgeons) was to close the abdomen and to refer the patient to a surgeon. That course of action, coupled with continued hemorrhage and the closest trained surgeon being more than one hour away, put the woman at substantial risk of immediate death. Therefore, the only viable, option was to try to stem the bleeding. When direct pressure and over-sewing the bleeding site failed, they decided to use an omental patch, similar to the procedure used to close perforated peptic ulcers. Neither physician had performed this procedure previously. A piece of healthy omentum (approximately 3cm X 3cm) was resected and placed over the bleeding surface (Figure 1). A 2-O silk suture with a small bore needle was used to tie the resected omentum over the ruptured nodule and to secure it at four equal points along the nodule's edge. The bleeding stopped and the abdomen was closed.

The patient recovered from surgery and was ambulating when she was transferred to the regional tertiary care hospital; she died 3 months later from cancer.

## Discussion

In resource-poor settings, evaluating a patient with a suspected acute hemoperitoneum challenges physicians’ resourcefulness. While ultrasound or computerized tomography is generally used in those settings with resources and adequate personnel, these modalities may not be available in rural or poor regions. In this case, the hospital's only ultrasound machine could not be moved and was located far from the emergency department. The closest CT scan was many hours away. The alternative was to do a diagnostic paracentesis, rather than a more formal peritoneal lavage (in either case, the immediate aspiration of ≥10mL non-clotting blood is positive.) The next diagnostic procedure in these urgent cases is to perform a laparotomy.

### Spontaneous Intraperitoneal Hemorrhage

The etiology of a spontaneous intraperitoneal hemorrhage in a fertile woman includes gynecologic, hepatobiliary, splenic, vascular, and other causes (Table 1) [1]. The most common of these is a ruptured ectopic pregnancy. The urgency of performing surgery increases with the scarcity of blood to transfuse and the relative lack of surgical experience of general medical officers without formal surgical training. In this case, the decision was initially made to use a Pfannenstiel incision, assuming that the patient had a ruptured ectopic pregnancy. Once they found that this was not the case, they did a formal midline laparotomy and encountered a life-threatening hemorrhage from a relatively unusual source—a hepatic cancer nodule.

**Table 1:** Differential diagnosis for hemoperitoneum in fertile woman

GYNECOLOGICAL	SPLENIC
Ectopic pregnancy	Trauma
Uterine leiomyoma/leiomyosarcoma	Spontaneous splenic rupture
Ruptured ovarian cyst/tumor	Iatrogenic
Endometriosis	Chronic myelomonocytic leukemia
Spontaneous uterine rupture	Infectious mononucleosis
	Vascular rupture/tumor
**VASCULAR**	**OTHER**
Ruptured splenic artery aneurysm	Gastric perforation
Ruptured cystic artery pseudoaneurysm	Ruptured intraabdominal neoplasm
Ruptured retroperitoneal varices	Ruptured pancreatic pseudocyst
Ruptured aorta/ connective tissue disorder	Disseminated intravascular coagulation
**HEPATIC/BILIARY**	
Trauma	
Hepatic adenoma/adenomatosis	
Hepatic primary/metastatic carcinoma	
Hemangioma	
Spontaneous rupture	
Hepatic angiomatosis	

### Omental Patch

Extrapolating from the information that they had learned, the physicians used an unconventional, but easily accomplished, approach to stem the bleeding - an omental patch. Omental patches, generally used to close duodenal ulcers, have also been used to repair seromuscular injuries to the colon [2], stem bleeding from elective hepatic carcinoma resections [3], and fill large hepatic cavities after removal of a hydatid cyst [4]. When used to seal a perforated gastroduodenal ulcer, the omentum is pulled through the perforation and fixed to the bowel to seal the "hole" created by the perforated ulcer. In this case, the operating physicians improvised, adapting a surgical procedure to an unusual situation to save a life. None of them had previously performed or seen this procedure, which requires no extra equipment. Their goal was to staunch the massive bleeding and to stabilize the patient so that she could be safely sent to a higher-level facility.

**Hepatocellula Carcinoma** It is unclear whether the hepatic tumors were primary hepatic carcinoma or metastatic lesions, since no pathology facilities were available. However, there was no evidence of other malignancy and primary hepatic carcinoma (hepatocellular and cholangiocarcinoma) is the 8^th^ most common malignancy in woman worldwide, with the population of many countries in sub-Saharan Africa being at higher risk due to early hepatitis B and C exposure and chronic infections, aflatoxins (*Aspergillus fumigates*) exposure, and alcohol use (>50-70g/day) [5]. As in this case, survival, even with treatment, is poor, with few patients surviving two years, even with aggressive treatment [6].

## Conclusion

Diagnosing and treating spontaneous intraperitoneal hemorrhages in resource-poor settings requires clinicians to respond rapidly, use innovative thinking, and maximize the skills and equipment they have at their disposal. Using an omental patch to stop massive localized intra-abdominal hemorrhage may be an important tool for all surgeons, not only those in remote settings.
